# A novel mutation of DNA2 regulates neuronal cell membrane potential and epileptogenesis

**DOI:** 10.1038/s41420-024-02029-9

**Published:** 2024-05-27

**Authors:** Yuting Liu, Haiyan Yang, Siyi Gan, Lu He, Rongrong Zeng, Ting Xiao, Liwen Wu

**Affiliations:** 1 Pediatrics Research Institute, The Affiliated Children’s Hospital of Xiangya School of Medicine, Hunan Children’s Hospital, Central South University, Changsha, Hunan China; 2 Department of Neurology, The Affiliated Children’s Hospital of Xiangya School of Medicine, Hunan Children’s Hospital, Central South University, Changsha, Hunan China; 3 Xiangya Hospital, Central South University, Changsha, Hunan China

**Keywords:** Genetics of the nervous system, Cell death

## Abstract

Mesial temporal lobe epilepsy (MTLE) is one of the most intractable epilepsies. Previously, we reported that mitochondrial DNA deletions were associated with epileptogenesis. While the underlying mechanism of mitochondrial DNA deletions during epileptogenesis remain unknown. In this study, a novel somatic mutation of DNA2 gene was identified in the hippocampal tissue of two MTLE patients carrying mitochondrial DNA deletions, and this mutation decreased the full-length expression of DNA2 protein significantly, aborting its normal functions. Then, we knocked down the DNA2 protein in zebrafish, and we demonstrated that zebrafish with DNA2 deficiency showed decreased expression of mitochondrial complex II–IV, and exhibited hallmarks of epileptic seizures, including abnormal development of the zebrafish and epileptiform discharge signals in brain, compared to the Cas9-control group. Moreover, our cell-based assays showed that DNA2 deletion resulted in accumulated mitochondrial DNA damage, abnormal oxidative phosphorylation and decreased ATP production in cells. Inadequate ATP generation in cells lead to declined Na+, K+-ATPase activity and change of cell membrane potential. Together, these disorders caused by DNA2 depletion increased cell apoptosis and inhibited the differentiation of SH-SY5Y into branched neuronal phenotype. In conclusion, DNA2 deficiency regulated the cell membrane potential via affecting ATP production by mitochondria and Na+, K+-ATPase activity, and also affected neuronal cell growth and differentiation. These disorders caused by DNA2 dysfunction are important causes of epilepsy. In summary, we are the first to report the pathogenic somatic mutation of DNA2 gene in the patients with MTLE disease, and we uncovered the mechanism of DNA2 regulating the epilepsy. This study provides new insight into the pathogenesis of epilepsy and underscore the value of DNA2 in epilepsy.

## Introduction

Mesial temporal lobe epilepsy (MTLE) is the most common form of focal and intractable epilepsy. It is frequently associate with hippocampal sclerosis [1, 2], characterized by neuronal loss and gliosis [1, 3, 4]. Over the past decades, our understanding of the underlying mechanisms leading to MTLE has improved significantly, especially mitochondrial functions and the genetic determinants during epileptogenesis attracted much attentions [5, 6]. Mutations of multiple genes and mitochondrial DNA deletions in brain tissue of epileptic patients were found through targeted gene sequencing and next-generation sequencing, including TSC, DEPDC5, PTEN, SLC35A2 and so on [7]. While the associations among genes mutations, mitochondrial deletions and epilepsy remain unclear.

Recent years, the associations between gene mutations and mitochondrial deletions have been found [8–12]. Several nuclear gene, like POLG, POLG2, TWNK, DNA2 and so on, are involved in mitochondrial DNA deletions. For example, mutations in POLG gene, caused early childhood mitochondrial DNA depletion syndromes or later-onset syndromes arising from mitochondrial DNA deletions [13, 14]. Mutations in TWINKLE caused a number of human disorders associated with mitochondrial dysfunction, neurodegeneration and premature ageing [15]. DNA2 gene mutations were recently discovered to be involved in mitochondrial DNA deletions and progressive muscular dystrophy [16–19]. These results highlight the associations between mitochondrial DNA deletions and mutations of nuclear genes.

Mitochondria are responsible for energy supply and calcium homeostasis in neuronal cells [20, 21]. Notably, about 40% of the total ATP generated by oxidative phosphorylation is consumed by the Na+, K+-ATPase to maintain Na+, K+ equilibrium through neuronal membranes, which is closely related to cell excitability. Importantly, it has been reported that inhibition of Na+, K+-ATPase activity caused epileptic seizures in rodents. And in chronic hypoxia of rat brain, the whole brain Na+, K+-ATPase activity was reduced by 77% [22–24]. These results suggested that Na+, K+-ATPase activity was related to neurodegenerative disease.

DNA2 is a conserved helicase/nuclease encoded by nuclear genome, and is widely expressed in the brain, heart, and muscle tissue. DNA2 mainly localizes in mitochondria, and responsible for DNA replication and DNA repair, maintaining the mitochondrial and nuclear DNA stability [25–28]. Some reports also showed that DNA2 gene mutations are associated with some diseases such as progressive extraocular paralysis and Seckel syndrome. Those patients were all found to carry multiple mitochondrial DNA deletions. And their muscle tissues were further sequenced, and 3 novel mutations in DNA2 gene were identified by whole-exome sequencing [16, 17, 29]. In another study, two intron mutations and one missense mutation in the DNA2 gene were identified in microcephalic primordial dwarfism patients by whole-exome sequencing, suggesting that mutations in the DNA2 gene may be associated with abnormalities in central nervous system development [16, 29]. These proved that DNA2 is closely related to neurological diseases, however the underlying pathway needs more investigations.

In this study, we identified a novel mutation of DNA2 gene in hippocampus of three MTLE patients, which caused significantly decreased levels of DNA2 protein in cells. We demonstrated that DNA2 absence can cause underdevelopment and epileptiform discharge signals in zebrafish model. And DNA2 deficiency resulted in declined ATP production, decreased the activity of Na+, K+-ATPase and change of cell membrane potential. These disorders inhibited neuronal cell differentiation and increased cell apoptosis. In conclusion, we provide mechanisms by which DNA2 regulate cell excitability and epilepsy.

## Results

### DNA2 is a clinical susceptible gene associated with epilepsy

**Fig. 1:**
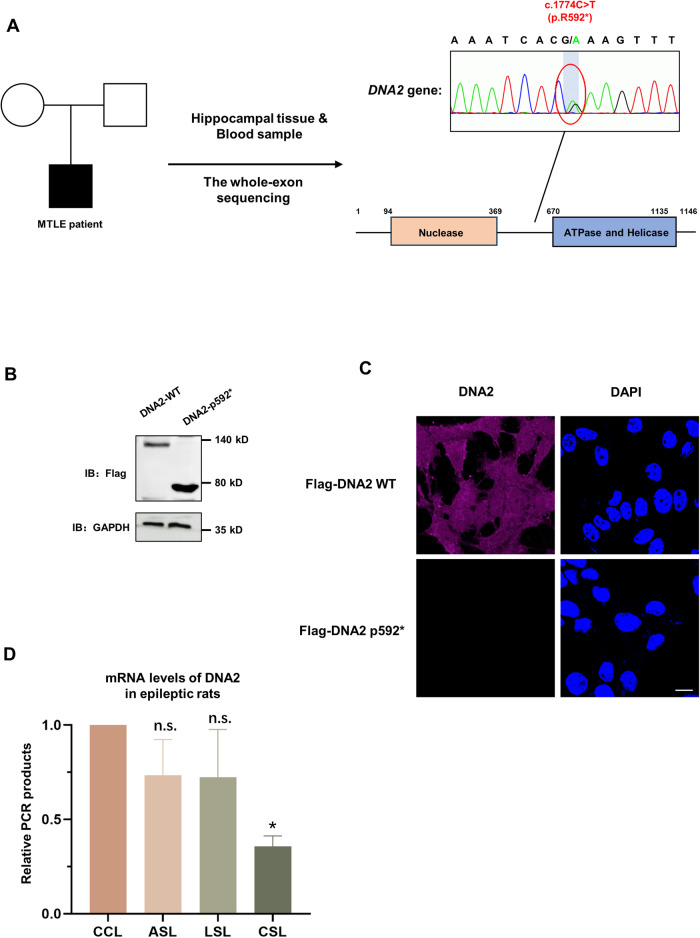
A novel mutation (p592*) of DNA2 was identified. **A** Genotyping of hippocampus tissue from MTLE patients by whole-exome sequencing. The right panel showed the sequencing results of the antisense strand of DNA2 gene. **B**, **C** The expression levels of FLAG-labeled WT or FLAG-labeled p592* mutant DNA2 in HEK293T cells analyzed by western blot and fluorescence. Scale bar, 10 μm. FLAG antibody (Huaxingbio, H1819) was used to test the DNA2 expression by western blot (**B**); and DNA2 antibody was used to label FLAG-WT or FLAG- P592* DNA2 (in purple) by fluorescence, nuclear DNA were stained with DAPI (in blue), scale bar: 10 μm (**C**). **D** RT-qPCR analysis of DNA2 transcriptional levels in epileptic rat models during normal stage, acute stage, chronic stage and latent stage (*n* = 2). *Y* axis represents the relative PCR products, *t*-test was used for comparison of two independent treatments (CCL and ASL; CCL and LSL; CCL and CSL). ASL acute sample from left hippocampus of epileptic rat, LSL latent sample from left hippocampus of epileptic rat, CSL chronic sample from left hippocampus of epileptic rat. Error bar represented SD, *p < 0.05, **p < 0.01, ***p < 0.001.

We have reported that mitochondrial DNA deletions in the hippocampal tissue of five MTLE patients [30]. By whole-exome sequencing, two of them were found carrying a mutation of DNA2 gene at site c.1774C > T in the hippocampal tissue (Fig. 1A). This mutation caused arginine at 592 of DNA2 to become a terminating amino acid (p.R592*). To date, this mutation of DNA2 gene has not been reported in disease. To figure out the effect of this mutation on the function of DNA2 protein, we first constructed FLAG-tagged wild-type DNA2 plasmid and FLAG-tagged R592* mutant DNA2 plasmid respectively, and transfected these plasmids into HEK293T cell line. The results showed that the R592* mutation caused decreased levels of full-length DNA2 protein as shown by western blot and fluorescence (Fig. 1B, C and Supplementary S1B), demonstrating that R592* mutation of DNA2 significantly decreased its full-length expression and affected its normal functions. Then, we checked the transcription levels of DNA2 in the pilocarpine rat model of temporal lobe epilepsy by RT-qPCR. And the transcription level of DNA2 also decreased significantly with the progression of epilepsy (Fig. 1D). These results suggested that this R592* mutation of DNA2, resulting in decreased DNA2 levels, may be associated with the pathogenesis of MTLE.

### DNA2 absence results in hallmarks of epileptic seizures in zebrafish

**Fig. 2:**
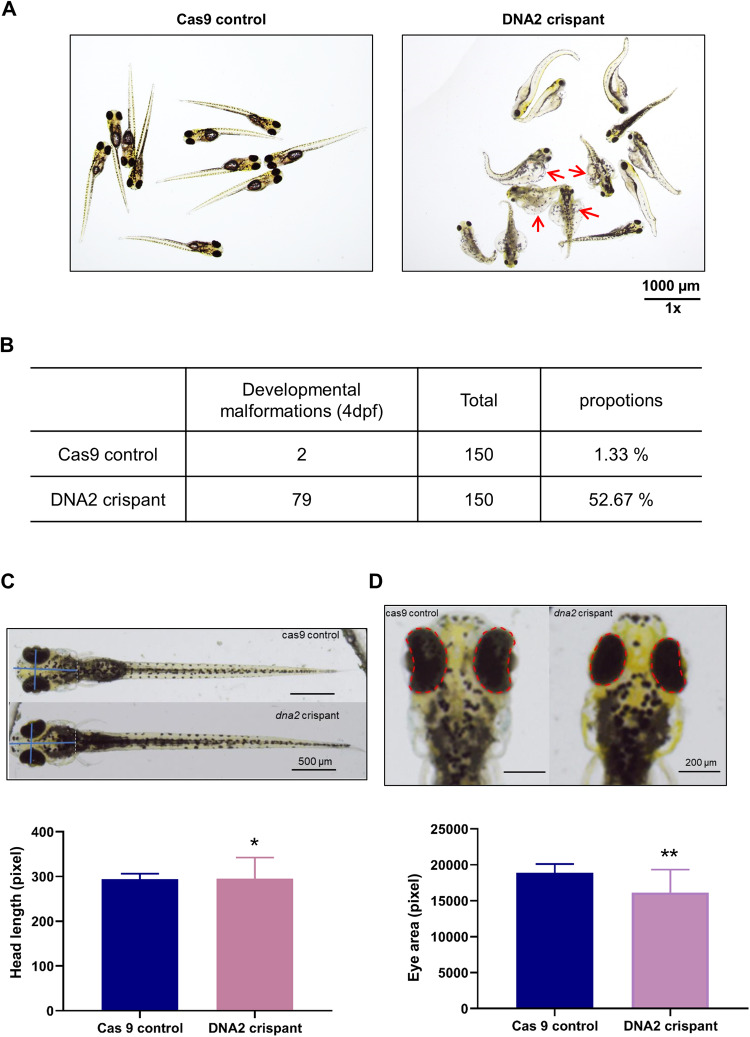
DNA2 deficiency affected the development and the behavior of zebrafish. **A** Representative image of normal zebrafish in Cas9 control group and zebrafish with developmental deformities in the DNA2-crispant group. Red arrows represented the dysplasia zebrafish. Scale bar: 1000 µm. **B** Number and ratios of zebrafish with developmental malformations at 4 days post fertilization in the Cas9 control group and DNA2 crispant group (*n* = 150). Representative images (upper panel) and quantification analysis (lower panel) of head length (**C**) and eye area (**D**) of zebrafish in the Cas9 control group (*n* = 20) and DNA2 crispant group (*n* = 20). *Y* axis represented head length in (**C**), and eye area in (**D**). Unpaired *t-*test was used here, and error bar represented SD, *p < 0.05, **p < 0.01, ***p < 0.001.

To verify DNA2 absence gets involved in the epileptogenesis, DNA2-knockdown zebrafish were generated by Crispant technique. The morphological changes and neuroelectrophysiology of zebrafish were analyzed. First, we counted the number of zebrafish with developmental deformities at 4 dpf (days post fertilization) in DNA2-knockdown group and Cas9-control group (it is difficult to distinguish whether dead individuals at 1–3 dpf are deformities). The malformation rate of zebrafish in the DNA2-knockdown group was significantly increased compared to the Cas9-control group (Fig. 2A, B). Then, we observed the morphological changes including eye distance, interocular area and body length. For each group, at least 20 zebrafish were analyzed (*n* > 20) and the number of zebrafish with morphological changes were counted. Compared to the Cas9-control group, the head length of zebrafish in DNA2-knockdown group is longer (Fig. 2C). Moreover, the interocular area of zebrafish showed significant difference (Fig. 2D), while the eye distance did not reach statistically difference between the Cas9-control zebrafish and DNA2-knockdown zebrafish.

**Fig. 3:**
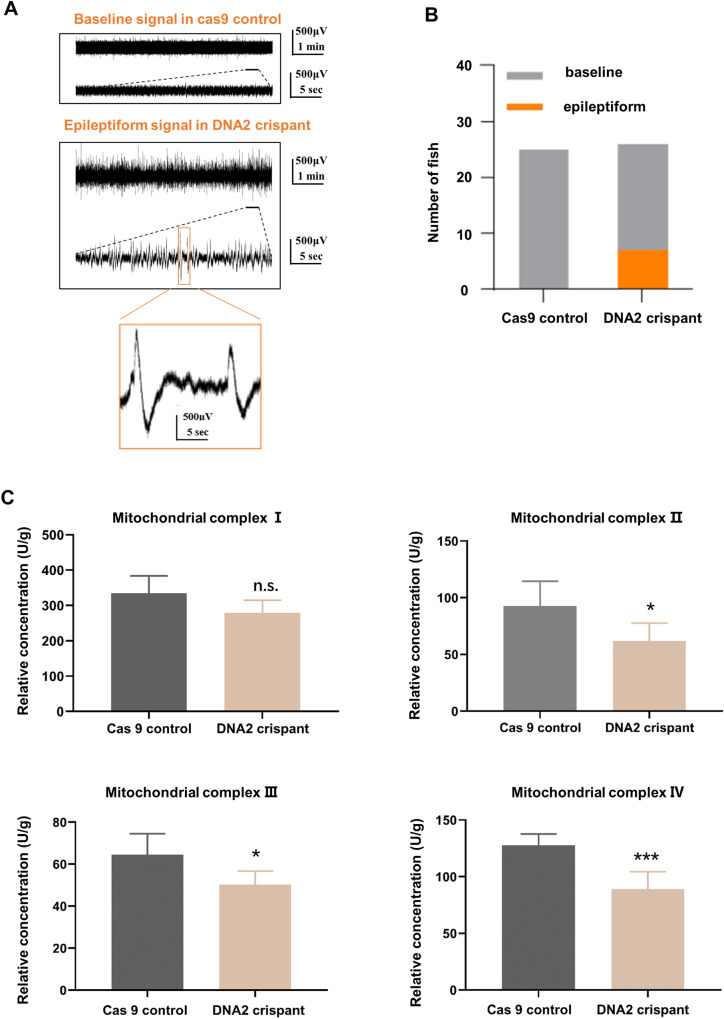
DNA2 absence decreased expression of mitochondrial complex II–IV and caused epileptic seizures in zebrafish. **A** Representative image of neuroelectrophysiological of zebrafish in the Cas9 control and DNA2 crispant group. **B** Histograms quantitative analysis of the result in (**A**). Number of zebrafish with epileptiform signal in brain were counted in the Cas9 control and DNA2 crispant group respectively. *Y* axis represented the number of fish. Chi-square test was used here (*p* value = 0.0052). **C** Analysis of the protein levels of mitochondrial respiratory complex in zebrafish hippocampus by ELISA experiment (*n* ≧ 3). *Y* axis represented relative concentration of mitochondrial complex in zebrafish. Unpaired *t-*test was used here, and error bar represented SD, *p < 0.05, **p < 0.01, ***p < 0.001.

Next, we determined the larval behaviors during development in these two groups of zebrafish. The spontaneous activity of individual larva was measured for 15 min, and the max acceleration and the moved distance did not reach statistically significance between these two groups of zebrafish. Also, the responses of zebrafish evoked by light/dark changes showed no significant difference in these two groups. While interestingly, we analyzed the neuroelectrophysiological of zebrafish and found that in Cas9-control group, all of the 25 zebrafish showed normal baseline signal, while in the DNA2-knockdown group, 7 of 26 zebrafish showed the epileptiform signal, and 19 of 26 zebrafish showed the normal baseline signal, which was significantly different from the Cas9-control group (Fig. 3A, B). Considering that DNA2 plays a role in mitochondrial DNA replication and repair, so we tested the effects of DNA2 deletion on the levels of mitochondrial respiratory complex. Our results showed DNA2 absence caused significantly decreased protein levels of mitochondrial respiratory complex II–IV (Fig. 3C). These results suggested DNA2 depletion caused epileptiform discharge signals through regulating the expression and function of mitochondrial respiratory complex in zebrafish brain. In summary, DNA2 loss affected the development and behaviors of zebrafish, especially neuroelectrophysiological of zebrafish, which further proved the key role of DNA2 played in epilepsy.

### DNA2 is important for mitochondrial oxidative phosphorylation and ATP production in cells

**Fig. 4:**
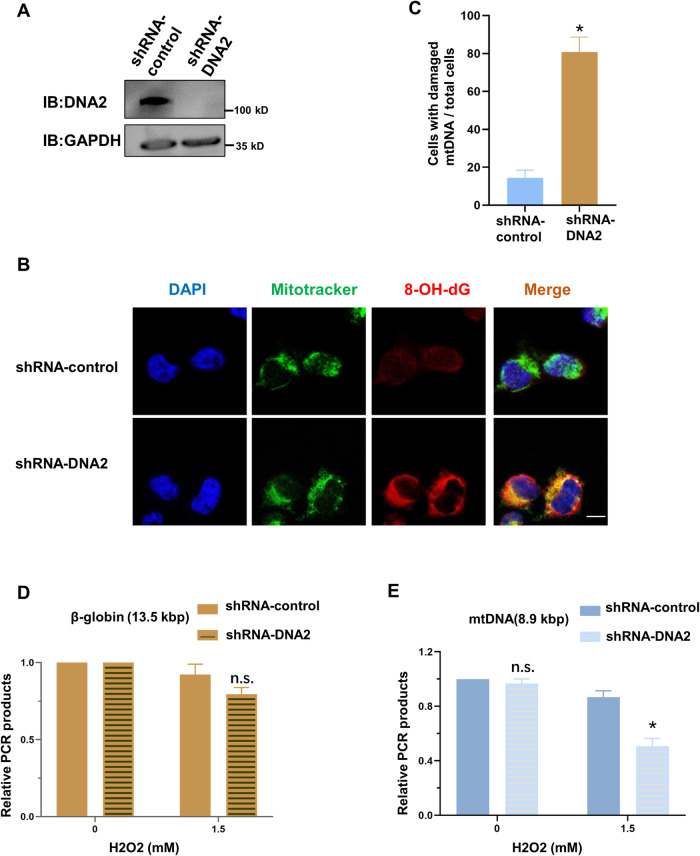
DNA2 depletion caused oxidative damage accumulated in mitochondrial DNA in SY5Y cells. **A** Western blotting analysis of DNA2 protein levels in shRNA-control and shRNA-DNA2 cells. DNA2 antibody was used to test the protein levels, and GAPDH antibodies serves as a loading control. **B** Immunofluorescence analysis of oxidative DNA damage in shRNA-control and shRNA-DNA2 cells. Representative images of cells with oxidative mitochondrial DNA damage. Mitochondria were labeled with mitotracker (in green), oxidative DNA damage were stained with anti-8-OHdG (in red), and nuclear DNA were stained with DAPI (in blue). Scale bar, 20 μm. **C** Histograms quantitative analysis of the result in (**B**). Histograms summarizing the ratios of cells with damaged mitochondrial DNA to total cell. A total of 150 cells were counted. *Y* axis represented the percentage of cells with oxidative damage to total cells. More than 50 cells were counted per experiment (*n* = 3 experiments), Unpaired *t-*test was used here, and error bar represented SD, *p < 0.05. H_2_O_2_ induced oxidative DNA damage in nuclei (**D**) and mitochondria (**E**). Histogram representing the amplification of a −13.5 kb nuclear DNA fragment or a −8.9 kb mitochondrial DNA fragment which was normalized to total amount of nuclear DNA or mitochondrial DNA copy number, following H_2_O_2_ treatment with different concentration. *Y* axis represented relative PCR products. Unpaired *t-*test was used here, error bar represented Mean with SD (*n* = 3), *p < 0.05, **p < 0.01, ***p < 0.001, n.s. means not significant statistically.

**Fig. 5:**
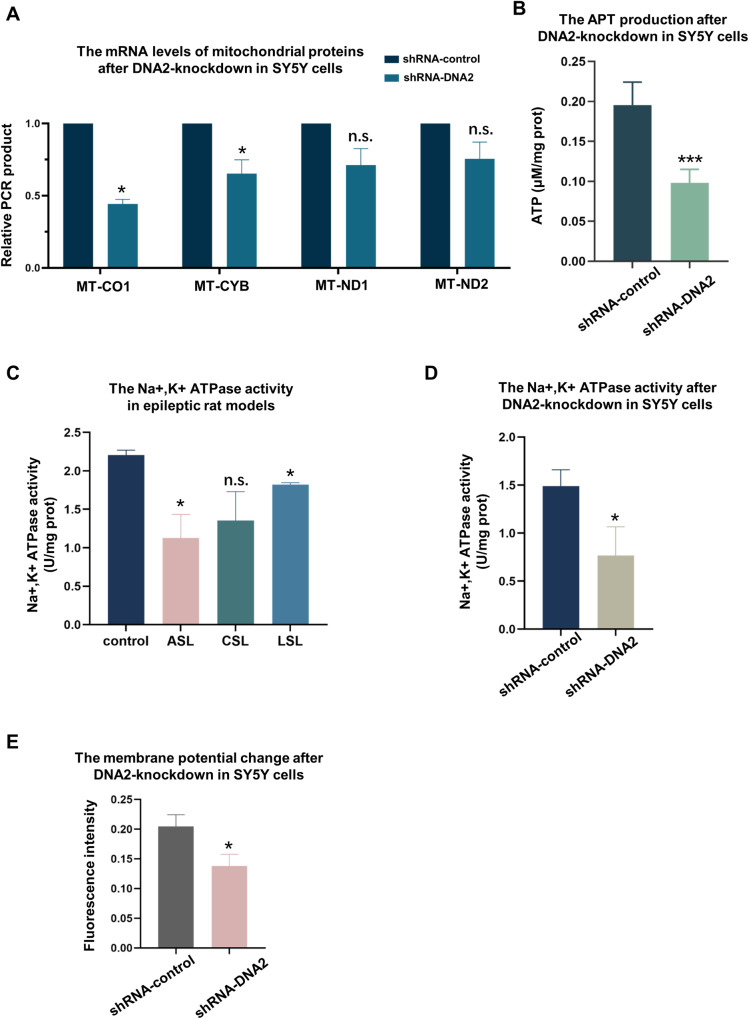
DNA2 knockdown affected ATP production, Na+-K+-ATPase activity and cell membrane potential in SY5Y cells. **A** Quantitative PCR analysis of transcriptional levels of genes encoded by mitochondrial DNA in shRNA-control and shRNA-DNA2 cells (*n* = 3). *Y* axis represented relative PCR products. Unpaired *t-*test was used here, and error bar represented SD, *p < 0.05, **p < 0.01, ***p < 0.001. **B** ATP production analysis in shRNA-control and shRNA-DNA2 cells by the ATP detection kit (*n* = 3). *Y* axis represented ATP production (μM/mg prot). Unpaired *t-*test was used here, and error bar represented SD, *p < 0.05, **p < 0.01, ***p < 0.001. Analysis of the Na+, K+-ATPase activity in hippocampal tissue from epileptic rat model (**C**) and in shRNA-control and shRNA-DNA2 cells (**D**). ASL acute sample from left hippocampus of epileptic rat, LSL latent sample from left hippocampus of epileptic rat, CSL chronic sample from left hippocampus of epileptic rat (*n* = 3). *Y* axis represented Na+, K+-ATPase activity (U/mg prot). Unpaired *t-*test was used here, and error bar represented SD, *p < 0.05, **p < 0.01, ***p < 0.001. **E** Analysis of cell membrane potential in shRNA-control cells and shRNA-DNA2 cells by cell membrane potential detection kit. Changes of fluorescence intensity suggest the polarization of the cell membrane. *Y* axis represented fluorescence intensity, and unpaired *t-*test was used here, and error bar represented SD, *p < 0.05, **p < 0.01, ***p < 0.001.

Next, we wanted to explore the pathway of DNA2 absence regulating epilepsy. Human DNA2 is required for mitochondrial DNA replication and repair. And our data showed DNA2 absence caused significant decreased protein levels of mitochondrial respiratory complex II–IV in zebrafish brain (Fig. 3C). Thus, we employed lentiviral transduction to knock down the expression of DNA2 in SH-SY5Y cells. Compared with shRNA-control group, the protein levels of DNA2 decreased under detection in shRNA-DNA2 group after 48 h transfection (Fig. 4A and Supplementary S4A). Then, these two groups of cells were treated with H_2_O_2_, and 8-OHdG antibody was used to measure the endogenous oxidative DNA damage, and mitotracker was used to label mitochondria in the cells. The results showed loss of DNA2 protein caused extensive 8-OHdG accumulation in the mitochondrial genome than in the nuclear genome in over 70% cells (Fig. 4B, C). We further employed the quantitative PCR assay, which can specifically amplify the 8.9 kb mitochondrial fragment and 13.5 kb nuclear fragment (β-globin) by designing specific primers, to test mitochondrial DNA and nuclear DNA damage occurred in the cells. Consistent with a previous study [31], more oxidative damage accumulated in mitochondrial DNA in shRNA-DNA2 cells than in shRNA-control cells. However, DNA2 knockdown had little effect on the amount of oxidative damage in nuclear DNA (Fig. 4D, E). As we all know, mitochondrial DNA encodes some key subunits of the mitochondrial respiratory chain complex, which are essential for ATP production in cells. So, we examined whether the deletion of DNA2 protein affected the expression of these proteins and ATP production. Our results showed that absence of DNA2 caused decreased transcription levels of some proteins in the electron transport chain by RT-qPCR, especially MT-CO1 (Mitochondrially Encoded Cytochrome C Oxidase I) and MT-CYB (Mitochondrially Encoded Cytochrome B) (Fig. 5A). Moreover, we analyzed the ATP production in cells using the ATP detection kit. Compared to shRNA-control cells, DNA2 deficiency led to significantly reduced ATP production in shRNA-DNA2 cells (Fig. 5B).

Together, our observations suggested that, DNA2 deficiency resulted in accumulated mitochondrial DNA damage and selectively reduced transcription levels of proteins encoded by mitochondrial DNA, further decreased the ATP production by affecting oxidative phosphorylation in cells.

### DNA2 deficiency regulates Na+, K+-ATPase activity and cell membrane potential

We have demonstrated that DNA2 depletion decreased ATP production in cells. Considering that about 40–50% of the ATP generated by the mitochondria are consumed by Na^+^, K^+^-ATPase to maintain Na^+^/K^+^ equilibrium and cell excitability [32], we sought to examine the association among DNA2 deletion, Na^+^, K^+^-ATPase activity and epilepsy in cells. First, we detected Na^+^, K^+^-ATPase activity of hippocampal tissue in the pilocarpine rat model of temporal lobe epilepsy. Interesting, we observed a decrease of the Na^+^, K^+^-ATPase activity in the hippocampal tissue of epileptic rats compared to normal rats, especially in the acute and chronic phases of epilepsy (Fig. 5C). This suggested the link of Na^+^, K^+^-ATPase activity and epileptogenesis. Then, shRNA-DNA2 cells and shRNA-control cells were collected and the Na^+^, K^+^-ATPase activity was tested using the Na^+^, K^+^-ATPase detection kit. Results showed that DNA2 knockdown significantly reduced the Na^+^, K^+^-ATPase activity in cells, as shown in Fig. 5D. These indicated that lack of DNA2 had effects on the Na^+^, K^+^-ATPase activity in cells.

Since Na^+^, K^+^-ATPase activity directly affects cell membrane potential and neuronal excitability, we detected the changes of cell membrane potential at the cellular level by the cell membrane potential detection kit. M09 in the kit is a lipophilic anionic fluorescent probe. Fluorescence will be detected when M09 probe enters into cells and binds to protein. Therefore, changes of fluorescence intensity suggest the polarization of the cell membrane. Here, compared to the shRNA-control cells, fluorescence intensity in the shRNA-DNA2 cells decreased significantly (Fig. 5E).

Taken together, these results implied that inadequate ATP supply caused by DNA2 absence regulated the Na+-K+-ATPase activity and cell membrane potential, and may further get involved in the epileptogenesis.

### DNA2 is necessary for neuronal cell differentiation and growth

**Fig. 6:**
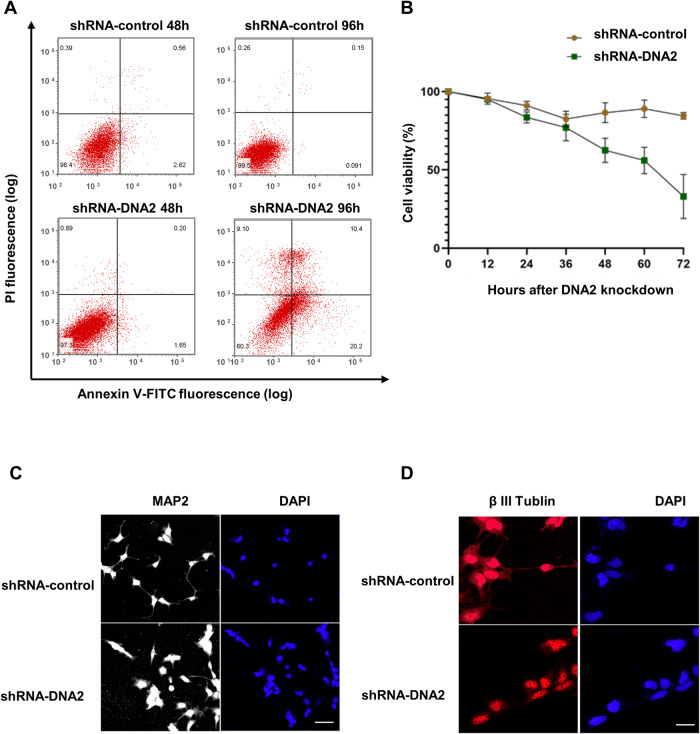
DNA2 is necessary for neuronal cell differentiation and growth. **A** Apoptosis analysis of shRNA-control cells and shRNA-DNA2 cells at 48 h and 96 h after cell transfection. *Y* axis represented PI fluorescence, and *X* axis represented Annexin V-FITC fluorescence. **B** Quantification of the percentage of viable cells in shRNA-control cells and shRNA-DNA2 cells. Cells were collected at the indicated time points and stained with trypan blue. Mean ± SD is shown for each time point from two independent biological replicates. **C**, **D** Immunofluorescence analysis of the effects of DNA2 loss on cell differentiation. Nuclear DNA were stained with DAPI (in blue), MAP2 (in white) and β tubulin III (in red) antibodies were used to label neuron-like cells. Scale bar, 20 μm.

Next, we investigated whether these disorders caused by DNA2 absence also affected differentiation and growth of neuronal cells during epileptogenesis. So, we performed flow cytometry (FACS) analysis to assess cell apoptosis using annexin V-FITC and PI staining after the depletion of DNA2 protein. The late apoptotic cells were clearly increased at 96 h after DNA2 was depleted (Fig. 6A). Considering that dead cells cannot be collected in the FACS assay, we also carried out the cell viability experiment to verify this result. The shRNA-control cells were viable, showing no significant change in the percentage of living cells within 48 h, while the shRNA-DNA2 cells begin showing significant cell death from 48 h (Fig. 6B). This is consistent with the FACS results. Moreover, the undifferentiated SH-SY5Y cells were induced to differentiate to a more mature and neuron-like cells, through addition of retinoic acid (RA) to the cell culture medium. Because the loss of DNA2 caused a large number of neuronal cells to die after 48 h, cells on the 4th day were collected to analyze the differentiation by immunofluorescence experiment. MAP2 and β tubulin III antibodies were used to label neuron-like cells. And results showed that absence of DNA2 inhibited the differentiation of SH-SY5Y to neuron-like cells (Fig. 6C, D).

In conclusion, we first demonstrated that DNA2 deficiency resulted in hallmarks of epileptic seizures in zebrafish brain. And cell-based assays illustrated the possible mechanism of DNA2 regulated epilepsy: DNA2 depletion caused accumulated mitochondrial DNA damage, affecting mitochondrial oxidative phosphorylation and ATP production. And further, mitochondrial dysfunction and insufficient ATP supply decreased Na^+^, K^+^-ATPase activity, leading to cell membrane potential change and inhibition of cell differentiation and growth during epileptogenesis.

## Discussion

Recent years, DNA2 gene mutations were discovered to be closely associated with progressive myasthenia and various neoplastic lesions [16]. Especially, in the congenital dwarfism with microcephaly disease, mutations in the exon of DNA2 gene were identified by whole-exome sequencing, suggesting DNA2 gene mutation may be related to the abnormal development of the central nervous system [29], but the underlying mechanism of DNA2 in the central nervous system remain unknown. In this study, we identified a novel pathogenic mutation of DNA2 gene in the hippocampus tissue of MTLE patients with multiple deletions of mitochondrial DNA, and this mutation significantly decreased the protein levels of DNA2 in cells. So, we knocked down DNA2 in cells and zebrafish to explore the relationship between the DNA2 mutation and epilepsy.

Previous studies have showed that DNA2 is a protein encoded by nuclear genes, mainly localized to mitochondria and involved in the replication and repair of mitochondrial DNA [25, 28]. In our research, we first examined the effects of DNA2 deletion on mitochondrial DNA oxidative damage, the transcription levels of protein encoded by mitochondrial DNA and the ATP production at the cellular level. Our data showed that DNA2 deficiency caused accumulated mitochondrial DNA damage in cells. These further resulted in declined transcription levels of several mitochondrial respiratory chain subunits encoded by mitochondrial DNA, and decreased intracellular ATP production. Together, DNA2 absence impaired mitochondrial electron transport chain by selectively damaging mitochondrial DNA, leading to inadequate ATP production. This dysfunction particularly impaired neuronal cells given their dependence on ATP to maintain membrane potential, propagate electrical signals and facilitate neuronal growth and differentiation.

Also, reports showed that about 40–50% of the total ATP is consumed by the Na^+^, K^+^-ATPase, and the disorder of Na^+^, K^+^-ATPase is associated with epilepsy and other central nervous system diseases [23, 24, 33]. Considering that Na^+^, K^+^-ATPase is responsible for ion homeostasis and cell excitability, we also detected a decrease in the activity of Na^+^, K^+^-ATPase enzyme in the epileptic rat model and DNA2-knockdown cells. These indicate that the change of energy metabolism in cells or tissues caused by mitochondria dysfunction also influence the Na^+^, K^+^-ATPase enzyme activity, and further affected ion homeostasis in cells. In order to determine whether there is a direct correlation between DNA2 and epilepsy, we constructed zebrafish model with DNA2 knockdown, and interestingly, the DNA2 knockdown zebrafish exhibited hallmarks of epileptic seizures, including abnormal development of the zebrafish, spontaneous activity, and epileptiform discharge signals of brain. It would be of great interest to further investigate the molecular participants during this process. Moreover, we found that the loss of DNA2 can inhibit the differentiation of SH-SY5Y cells to neuronal-like cells, and lead to increased cell apoptosis. These data suggested that DNA2 absence may regulate neuronal death in epilepsy. While the underlying pathway need more investigation.

In summary, DNA2 mutations are frequently reported with neuromuscular disease, little is known about the role of DNA2 in central nervous system, especially in epilepsy. Our study is the first to identify the DNA2 variant (pR592*) in the hippocampal tissue of MTLE patients, and moreover, we explored the possible mechanism underlying the involvement of DNA2 in epilepsy. Our results indicated that DNA2 absence regulated cell membrane potential and cell growth through regulating ATP generation by mitochondrial oxidative phosphorylation and Na^+^, K^+^-ATPase activity, thus getting involved in epilepsy. Importantly, these data provide new insight into the pathogenesis of epilepsy and underscore the value of DNA2 in epilepsy.

## Materials and methods

### Plasmids and DNA sequences

See Table 1 for plasmids and DNA sequences (oligos, primers) used in this study.

**Table 1:** Plasmids and DNA sequences (oligos, primers) used in this study.

Oligonucleotides	sequence
DNA2 shRNA F’	GCCAGGAGATATCATTCATTT
DNA2 shRNA R’	AAATGAATGATATCTCCTGGC
DNA2 (WT) -F‘	AGGTCGACTCTAGAGGATCCCGCCACCATGGAGCAGCTGAACGAACTGG
DNA2 (WT) -R‘	TCCTTGTAGTCCATACCTTCTCTTTGAAAGTCACCCAATATG
DNA2 (R592*) -F‘	AGGTCGACTCTAGAGGATCCCGCCACCATGGAGCAGCTGAACGAACTG
DNA2 (R592*) -R‘	TCCTTGTAGTCCATACCAAGTTTTTTGCTGACAAACGTGTTTTC
MT-CO1-1F	CCTACTCCTGCTCGCATCTG
MT-CO1-1R	AGAGGGGCGTTTGGTATTGG
MT-CYB-1F	TGGCTGAATCATCCGCTACC
MT-CYB-1R	TCCCAATGTATGGGATGGCG
MT-ND1-1F	CAACATCGAATACGCCGCAG
MT-ND1-1R	AATCGGGGGTATGCTGTTCG
MT-ND2-1F	AGCACCACGACCCTACTACT
MT-ND2-1R	TGGTGGGGATGATGAGGCTA

### Epileptic rat model

Epileptic rat models were constructed following the established protocol by our lab previously [3]. In brief, 82 of 21-day-old (which is similar to the onset age of MTLE patients) Sprague–Dawley rats weighing 30–50 g (clean grade, provided by Hunan Children’s hospital) were acquired and were randomly allocated to cages. The rats were allowed to adapt to laboratory conditions for at least 3 days before the experiments were begun. Then, 82 male rats were randomly divided into a control group of 30 rats and a model group of 52 rats (Control/Acute seizure group: 10/12; Control/Latent seizure group: 10/12; Control/Chronic seizure group 10/12.): (1) The model rats were injected pilocarpine hydrochloride (50 mg/kg, 50 mg/kg, i.p. Sigma-Aldrich Inc.), and status epilepticus (SE) occurred 18–20 h after pilocarpine hydrochloride induction; (2) After intraperitoneal injection of pilocarpine, the rats gradually developed sweating, tearing, mechanical chewing, scratching, continuous nodding forelimb clonus and so on. (3) The severity of seizures was determined according to Lado seizure grading criteria. And The seizures were stopped by giving 100 mg/ml chloral hydrate immediately after epileptic status lasted at least 40 min (often, Lado standard level 5–7: epileptic status lasted at least 40 min) or the rats were dying; (4) Rats that did not meet the Lado standard level 5–7 and died of anesthesia or convulsion with 100 mg/ml chloral hydrate were excluded from the experiment. While, rats that met the criterion were subjected to follow-up experiments.

### ELISA experiment and quantification

The protein levels of mitochondrial complex I–IV were tested by the ELISA kit for Zebrafish Mitochondrial Respiratory chain complex (MM-91905O1, MM-91907O1, MM-91910O1, MM-91913O1). First, brain tissues of zebrafish were homogenized, and the supernatant of brain tissue were place in the ELISA plate. Then, experiments were done following the instructions. Finally, the protein concentration of mitochondrial complex I–IV were calculated by the measured OD value, according to the standard curve.

### H_2_O_2_ treatment and quantitative PCR

SY5Y cells were transfected with shRNA-control or shRNA-DNA2. Forty-eight hours after transfection, cells were treated with H_2_O_2_ for 30 min. Then, cells were harvested and nuclear or cytoplasmic DNA was isolated with DNA extraction kit (TIANGEN, DP304). Then, the relative amounts of H_2_O_2_-induced DNA damage were tested by quantitative PCR as previously described [31]. In brief, a 8.9 kbp (mitochondrial DNA fragments) or a 13.5 kbp (nuclear DNA fragments) was amplified by PCR. A 221-bp fragment of mitochondrial DNA was amplified to determine the copy number of mitochondria DNA (It is known that mitochondrial DNA vary in different cells or tissues, depending on the energy requirements. Thus, the 221-bp mitochondrial DNA was designed to rule out the fluctuation in the copy number of mitochondrial DNA). And the PCR products were quantified by PicoGreen, and relative PCR products, representing relative level of oxidative DNA damage, was calculated by dividing the fluorescence value (OD value) of the treated samples by that (OD value) of untreated samples.

For the quantification of PCR products by PicoGreen, 10 µl PCR products were added to 90 µl of 1×TE buffer, then, mixed with 100 µl PicoGreen reagent. These solutions were incubated in dark, room temperature for 15 min, and the fluorescence (OD value) were read on the Microplate Reader.

### Cell culture

Unless otherwise indicated, cells were incubated at 37 °C in 5% CO_2_. SH-SY5Y cells (purchased from Abiowell, AW-CCH335) were cultured in Dulbecco’s modified Eagle’s medium (DMEM; Sigma, St. Louis, MO) containing 15% fetal bovine serum, FBS (VivaCell, Shanghai, China) and 1% penicillin-streptomycin.

### Virus production and infection

Briefly, 293T cells were transfected by using Lipo3000 (Invitrogen L3000008). Virus was collected 72 h posttransfection, and infections were carried out in the presence of Polybrene (Servicebio, G1803).

### Plasmids construction and cell transfection

To knock down the DNA2 levels in cells, the shRNA-DNA2 sequence was cloned into pCDH-CMV-MCS-EF1-copGFP plasmid, and was transfected into HEK293 cells together with the pMD2G and pSPAX.2 plasmids using Lipo3000 (Invitrogen, L30000008) according to the manufacturer’s instructions. The virus was collected at 72 h posttransfection and infected into SH-SY5Y cells.

### Immunofluorescence and microscopy

For immunofluorescence assay, cells were grown on glass coverslips, washed twice with PBS and then fixed with 4% paraformaldehyde for 20 min at room temperature. cells were permeabilized with 0.1% Triton X-100 in PBS for 10 min following washed with PBS for three times. Next, cells were blocked with 5% BSA in PBS for 1 h at room temperature and incubated with primary antibodies overnight at 4 °C (DNA2 antibody: ABclonal, AP354; Mitotracker: Beyotime, C1049B; 8-OH-dG: Bioss, BR1278R). Next day, cells were washed with PBST for three times and incubated with fluorescence conjugated secondary antibodies at 37 °C for 1 h (Servicebio: Alexa Fluor 488-conjugated Goat anti Mouse IgG, GB25301; Cy3 conjugated Goat Anti-Rabbit IgG, GB21303). Finally, cells were stained with antifade mounting medium with DAPI, and further analyzed in the laser scanning confocal microscopy.

### ATP production assay

Cells were collected and the ATP production were tested using the ATP assay kit (Beyotime, S0026). According to the manufacturer’s instructions. Briefly, cells were lysed and centrifugated to remove cell debris, and the supernatant was added to the substrate solution following the manufacturer’s instructions. The luminescence was recorded in an Illuminometer.

### Na+, K+-ATPase activity assay

The detection of Na^+^, K^+^-ATPase activity was performed using Na^+^, K^+^-ATPase activity assay kit purchased from Solarbio (BC0065). Cells or tissues were lysed and supernatant was collected and added the solutions in the kit following the manufacturer’s instructions. The luminescence was recorded in an Illuminometer.

### Cell apoptosis assay

Cells were assayed for apoptosis with the Annexin V-FITC Apoptosis Detection Kit (Beyotime, C1062S) and detected by flow cytometry according to the manufacturer’s protocol. Cell samples were analyzed using a FACSCalibur cytometer (BD Biosciences, USA).

### Cell viability assay

The shRNA-DNA2 and shRNA-control cells were seeded at 1.5 × 10^5^ cells/well in a 6-well plate. Cells were collected at the indicated time points, stained with trypan blue (Corning, 25-900-CI), and counted on a hemocytometer to calculate the percentage of viable cells out of the total cells based on trypan blue uptake.

### Cell membrane potential test

The test of cell membrane potential change was carried out using the membrane potential test kit (Biossen, BES20038BO). Cells were grown on the coverslip and the fluorescence probe was added following the instructions. Then, the luminescence was recorded in an Illuminometer.

### CRISPR/Cas 9 zebrafish

To generate CRISPR/Cas9-mediated DNA2 knockdown zebrafish, we injected the sgRNA with recombinant Cas9 protein into one-cell stage zebrafish embryos. The injected solution contained 90 ng/µl sgRNA, 250 ng/µl Cas9 protein in a single mix.

### Morphology observation

Five zebrafish larvae (5 dpf: day post fertilization) were selected and placed in 18-well plates. Adjusting the position of the zebrafish to ensure that the zebrafish is horizontal with its back facing up on the display screen. The picture was taken and stored by Nikon SMZ800N stereo microscope. ImageJ was used to measure eye distance and body length and intereye area of zebrafish in the images.

### Behavior test

Five dpf zebrafish larva were selected and put into the 96-well plate. The zebrafish larva was first allowed to adapt to the dark environment for 30 min, and then spontaneous behavior was carried out by the behavior trajectory tracking system. After that, the zebrafish larva was directly received the dark and light stimulation. There were four cycles of stimulation, 5 min of darkness/brightness in each cycle and the zebrafish larva behavior tracks were collected.

### Electrophysiological test

Zebrafish larvae (5–6 dpf) were put into the sample plate, and were anesthetised by adding 300 μM pancuronium bromide (Sigma) E3 solution. After 3 min, the larvae were fixed with back embedded with 2% low melting point agarose gel (Sangong). Then, E3 culture solution was added to cover the agaroses and reference electrodes. Glass microelectrodes were used from the visual cap to measure the local field potential of the brain by adding 2 M sodium chloride solution to the glass microelectrode. Electrophysiological signal amplifiers (A-M Systems) with high impedance were used to record electrical signals experimentally via an analog-to-digital converter. Measurement Computing used digital processing of electrical signal, sampling frequency was 10 kHz, and filter was set as 1 Hz~5 kHz, the measurement time was 15 min, the data were collected and analyzed using DClamp open source software.

### Animal studies

The sample size of animal studies was estimated based on the results of in vitro pre-tests on animals and previous experience. And randomization was used for animal studies.

### Quantification and statistical analysis

#### Statistical analysis

The data in this study are presented as the mean ± standard deviation (SD). Statistical analyses were carried out with Student’s *t* test for comparison of two independent treatments. Values were considered significant when *p* < 0.05. The statistical program GraphPad Prism 7.00 (GraphPad Software, Inc., USA) was used to perform all the analyses. For all tests, *p < 0.05; **p < 0.01; ***p < 0.001 (Student’s *t* test). n.s., not significant statistically.

### Supplementary information

Figure S1B

Figure S4a

original data attached to S1B

original data attached to S4A

## Data Availability

The datasets used and/or analyzed during the present study are available from the corresponding author on reasonable request.
