# Supplementary figures and images for: A novel mutation of DNA2 regulates neuronal cell membrane potential and epileptogenesis
Source: Cell Death Discov. 2024 May 27;10:259. doi: 10.1038/s41420-024-02029-9 (PMC11130173; doi:10.1038/s41420-024-02029-9)

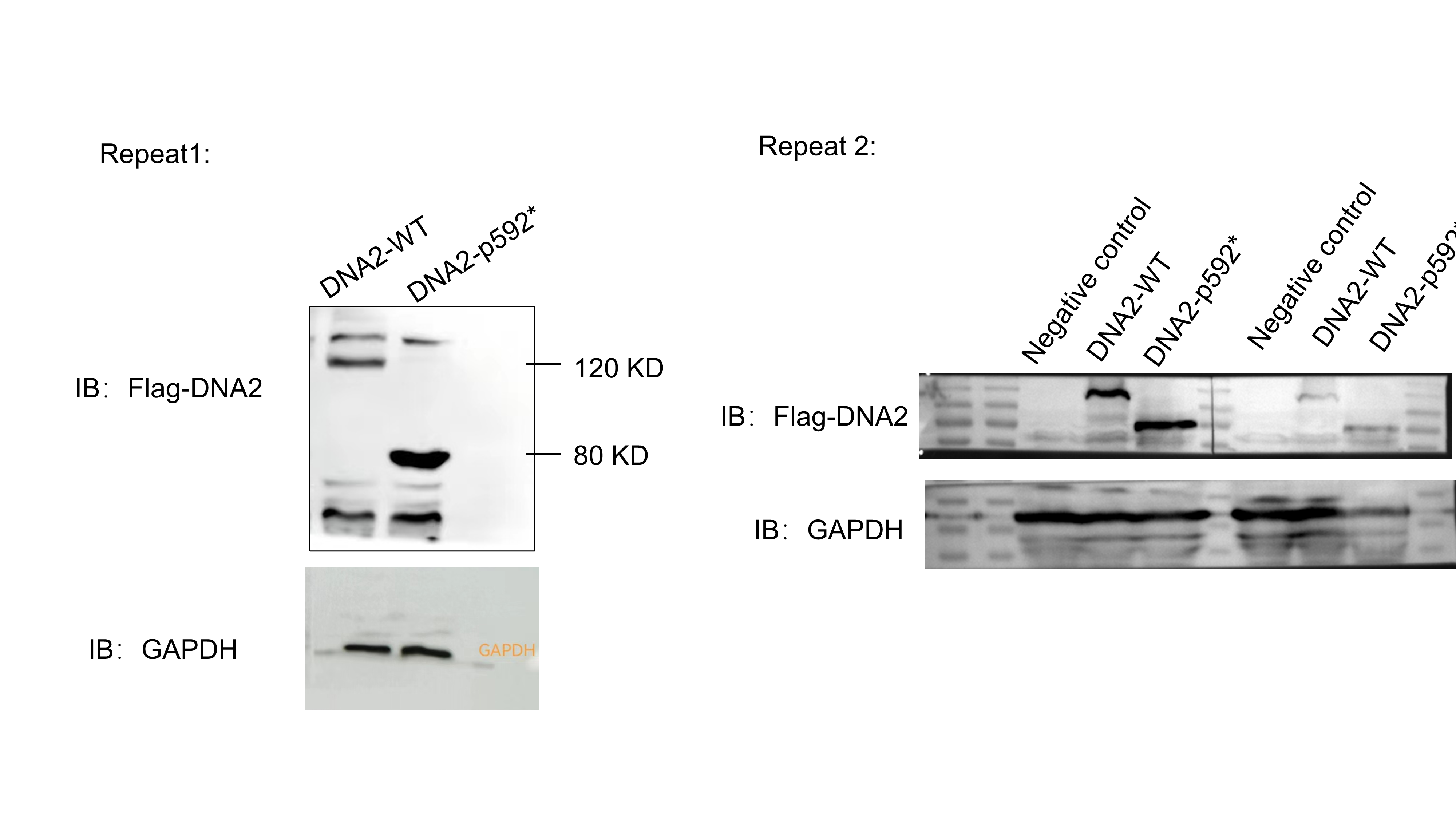

Supplement: Supplementary file 1 — Figure S1B [file 41420_2024_2029_MOESM1_ESM.png]

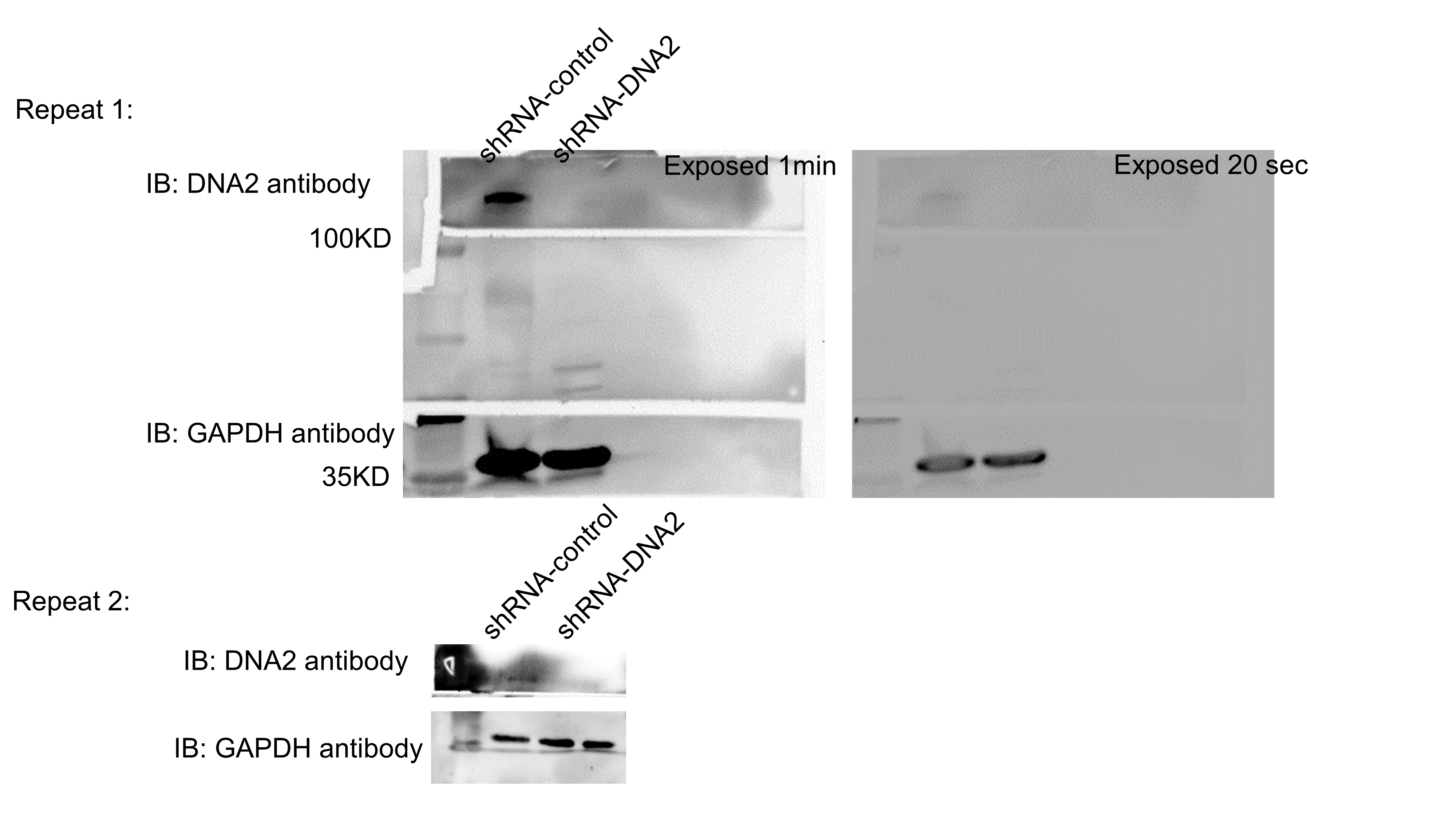

Supplement: Supplementary file 2 — Figure S4a [file 41420_2024_2029_MOESM2_ESM.png]
